# CYP2C19 and CYP2J2 genotypes predict praziquantel plasma exposure among Ethiopian school-aged children

**DOI:** 10.1038/s41598-024-62669-w

**Published:** 2024-05-22

**Authors:** Tigist Dires Gebreyesus, Eyasu Makonnen, Nigus Fikrie Telele, Abbie Barry, Rajabu Hussein Mnkugwe, Heran Gerba, Marja-Liisa Dahl, Eleni Aklillu

**Affiliations:** 1 Department of Global Public Health, Karolinska Institutet, Karolinska University Hospital, Tomtebodavägen 18A, 171 77 Stockholm, Sweden; 2 Ethiopian Food and Drug Authority, Addis Ababa, Ethiopia; 3 Center for Innovative Drug Development and Therapeutic Trials for Africa, College of Health Sciences, Addis Ababa University, Addis Ababa, Ethiopia; 4 Department of Pharmacology and Clinical Pharmacy, College of Health Sciences, Addis Ababa University, Addis Ababa, Ethiopia; 5 Clinical Pharmacology, Department of Laboratory Medicine, Karolinska Institutet, Karolinska University Hospital, Stockholm, Sweden; 6 Department of Clinical Pharmacology, School of Medicine, Muhimbili University of Health and Allied Sciences, Dar es Salaam, Tanzania

**Keywords:** *CYP2C19*, *CYP2J2*, *CYP3A5*, Praziquantel, Plasma concentration, Schistosomiasis, Ethiopia, Metabolism, Genetic association study, Public health, Infectious diseases

## Abstract

Metabolism of praziquantel (PZQ), a racemic mixture and the only drug approved to treat *S. mansoni* infection, is mediated by genetically polymorphic enzymes. Periodic school-based mass drug administration (MDA) with PZQ is the core intervention to control schistosomiasis. However data on the impact of pharmacogenetic variation, nutrition, and infection status on plasma PZQ exposure is scarce. We investigated genetic and non-genetic factors influencing PZQ plasma concentration and its metabolic ratios (*trans*-4-OH-PZQ/PZQ and *cis*-4-OH-PZQ/PZQ). Four hundred forty-six school children aged 7–15 years from four primary schools in southern Ethiopia who received albendazole and PZQ preventive chemotherapy through MDA campaign were enrolled. Genotyping for common functional variants of *CYP3A4 * (*1B)*, CYP3A5 * (*3, *6)*, CYP2C19 * (*2, *3, *17)*, CYP2C9 * (*2, *3), and CYP2J2*7 was performed. Plasma concentrations of PZQ, *trans*-4-OH-PZQ, and *cis*-4-OH-PZQ were quantified using UPLCMS/MS. Carriers of *CYP2C19* defective variant alleles (*2 and *3) had significantly higher mean PZQ plasma concentration than CYP2C19*1/*1 or *17 carriers (p = 0.005). CYP2C19*1/*1 and CYP2C19*17 carriers had higher *trans*-4-OH-PZQ/PZQ and *cis*-4-OH-PZQ/PZQ metabolic ratios compared with CYP2C19*2 or *3 carriers (p < 0.001). CYP2J2*7 carriers had lower mean PZQ plasma concentration (p = 0.05) and higher *trans*-4-OH-PZQ/PZQ and *cis*-4-OH-PZQ/PZQ metabolic ratios. Male participants had significantly higher PZQ concentration (p = 0.006) and lower metabolic ratios (p = 0.001) than females. There was no significant effect of stunting, wasting, *S. mansoni* or soil-transmitted helminth infections, *CYP3A4*, *CYP3A5*, or *CYP2C9* genotypes on plasma PZQ or its metabolic ratios. In conclusion, sex, *CYP2C19* and *CYP2J2* genotypes significantly predict PZQ plasma exposure among Ethiopian children. The impact of *CYP2C19* and *CYP2J2* genotypes on praziquantel treatment outcomes requires further investigation.

## Introduction

Globally, about 250 million people are currently infected with schistosomiasis, and 800 million are at risk of infection in endemic areas, mainly in tropical and sub-tropical regions^1–3^. Over 90% of the disease burden is from Sub-Saharan African (SSA) countries^4^. In Ethiopia, approximately 38.3 million people live in schistosomiasis-endemic areas^5^. Schistosomiasis was first reported in the country in 1934^6^, and children in high transmission areas are the most affected. Repeated exposure to contaminated water with infectious cercariae leads to chronic schistosomiasis, especially in children^7^. In Ethiopia, schistosomiasis remains among the significant causes of morbidity in children^8^. Chronic schistosomiasis can lead to several health complications, including malnutrition, anemia, impaired childhood development, fatigue, exercise intolerance, and poor cognitive function^9–11^.

Preventive chemotherapy using mass praziquantel (PZQ) administration, targeting school-aged children, is the cornerstone prevention and control strategy recommended by the World Health Organization (WHO) to control schistosomiasis and interrupt transmission in endemic regions^12^. PZQ has been used in large-scale mass drug administration (MDA) programs worldwide since 1984^12^. The standard recommended dosage for treating schistosomiasis is 40 mg/kg body weight. However, this standard dosage resulted in varying treatment outcome (efficacy and safety) findings in SSA among pre-school and school-aged children^13^. Variability in drug response may result from various factors, including genetic variations, nutrition, environmental factors, and type of disease^14^.

Genetic variations influence variability in drug exposure and treatment response of various infectious diseases^15–17^. However, data on the importance of genetic variation for variability in PZQ plasma exposure between populations and individuals is scarce^18–21^. Assessing the importance of genetic and non-genetic factors that influence variation in PZQ exposure in genetically diverse populations of Africa, where the disease is most prevalent, is imperative^22^ to optimize treatment^23^. Recent studies highlighted the significant contribution of genetic variations on PZQ plasma concentration and treatment outcomes^20,24^ and the need for pharmacogenetic studies of PZQ, especially in the African continent^19^.

Praziquantel, a racemic mixture of the biologically active enantiomer (R-PZQ) and distomer (S-PZQ)^18,19^, is metabolized in the liver by CYP enzymes, including *CYP1A2, CYP3A4, CYP3A5, CYP2C9, CYP2C19* and *CYP2D6*^19,25^. The two major metabolites in humans are *trans-*4-OH-PZQ and *cis-*4-OH-PZQ^18,19,25,26^. The CYP enzymes are genetically polymorphic, showing broad inter-individual variability in enzyme activity, which may affect PZQ plasma concentration^27^. In this study, we assessed the effect of genetic variations on plasma concentrations of PZQ and its major metabolites (*trans-* and *cis-*4-OH PZQ) among school children who received mass PZQ and albendazole (ALB) in two rural districts in Southern Ethiopia.

## Methods

### Study design, area, and population

This prospective observational pharmacogenetics-pharmacokinetics study was conducted to investigate the effect of genetic variations on plasma concentrations of PZQ and its major metabolites. The study was conducted in two rural districts in southern Ethiopia in January 2019. The two districts, Hawella Tula and Wondo Gennet, are located around the shore of Lake Hawassa and are known for their high endemicity of schistosomiasis and soil-transmitted helminths^5,28^.

The study population consisted school children who were eligible for PZQ and ALB combination preventive chemotherapy for the control of schistosomiasis and soil-transmitted helminths as per the WHO and the Ethiopian NTD preventive chemotherapy guidelines. A total of 446 schoolchildren aged 7–15 years attending four primary schools-Bushulo, Kidus Pawulos, Finchawa, and Wosha-located in the two study districts were enrolled in this study. All children attending the study schools were eligible for PZQ and ALB preventive chemotherapy.

### Ethical considerations

Prior to the study initiation, permission to conduct the study was obtained from regional, zonal, and woreda Health and Education offices. The study received ethics approval from Southern Nations, Nationalities and Peoples Region Health Bureau ethical clearance committee (Ref no 902-6-19/14966), The Ethiopia National Research Ethics Review Committee (Ref no MoSHE//RD/141/9848/20), and The Swedish Ethics Review Authority (Ref No 2020-00845). Study participants and their guardians/parents were informed about the study and data collection processes, and children whose parents/guardians gave written informed consent were enrolled in the study. The study was conducted according to the guidelines of the Declaration of Helsinki. Informed consent from parents/guardians and assent from participants who were > 12 years of age were obtained.

### Data collection and preventive chemotherapy

Socio-demographic characteristics, including sex, age, and clinical data such as body weight, height, nutritional status, and infection status, were collected using a case record form through interviews before MDA. School-based MDA with PZQ and ALB 400 mg was given as preventive chemotherapy through an MDA campaign led by the national NTD control public health program coordinators. The study team had no role in the MDA planning, implementation, and administering of the drugs. The dosing of PZQ was based on the WHO dose pole based on height (corresponding to 40 mg/kg body weight)^29^. All children attending primary schools in the two rural districts received MDA irrespective of their infection status. The nutritional status of the participants was assessed by converting the height for age Z score (HAZ) for stunting and BMI for age Z score (BAZ) for wasting using WHO Anthro Plus software^30^.

### Stool and blood samples collection

Two weeks before MDA, stool samples were collected for microscopic examination for screening and diagnosis of *S. mansoni* and soil transmitted helminths infection using Kato-Katz technique. On the MDA Day, whole blood samples for genotyping were collected in EDTA-containing vacutainer tubes from 446 participants and stored at −80 °C. Two hours post-dose, blood samples were collected from each participant in the heparinized tube and centrifuged at 1000 rpm for 10 min, and plasma samples were stored at −80 °C until analysis. Both whole blood and plasma samples were packed with dry ice and shipped to Karolinska Institutet, Stockholm, Sweden, for laboratory analysis.

### Quantification of plasma PZQ, *trans*-and *cis*-4-OH-PZQ concentration

Reference standards such as rac-PZQ, *trans*-4-OH-PZQ, and *cis*-4-OH-PZQ and their respective internal standards rac-PZQ-d11, *trans*-4-OH-PZQ-d5, and *cis*-4-OH-PZQ-d5 were purchased from Toronto Research Chemicals (Toronto, Ontario, Canada). Acetonitrile, methanol, and formic acid of mass spectrometry (MS) grade were purchased from Merck (Darmstadt, Germany). Ultra-pure MilliQ water was prepared using a Milli-Q water purification system (Merck Millipore, Massachusetts, USA). Blank plasma was kindly supplied by the blood bank of the Karolinska University Hospital Huddinge (Stockholm, Sweden).

The plasma sample preparation procedure for quantification of PZQ, *trans*-4-OH-PZQ and *cis*-4-OH-PZQ was adapted from Nyla et al*.*^25^ with slight modification. Briefly, 100 µL of plasma sample was added to 300 µL of internal standard solution consisting of 50 ng/mL of rac-PZQ-d11, *trans*-4-OH-PZQ-d5 and *cis*-4-OH-PZQ-d5 in a 50:50 mixture (v:v) of acetonitrile: methanol. The mixture was vortexed for 3 min, followed by centrifugation for 20 min at 3220*g* at 4 °C. Then, 75 µl of the supernatant was diluted with 75 µL MilliQ water and 5 µL was injected into the UPLC-MS/MS system. Standards and quality control (QC) samples were prepared in the same manner by adding 10 µL standard and QC 10× concentrated solutions to 90 µL blank plasma and precipitating as above.

The UPLC–MS/MS method of quantification of the analytes was done as described previously^20,31^. The calibration curves were constructed within the range of 2.4 to 2500 ng/mL for PZQ and *cis*-4-OH-PZQ, and from 24 to 25,000 ng/mL for *trans*-4-OH-PZQ, since the levels of *trans-*4-OH-PZQ were very high in the samples. About 7–9 calibration points were injected twice before and after the samples. The QC samples were injected every 20 samples. The analytes were quantified using the analyte to internal standard integrated peak area ratio with the Mass Lynx application manager Target Lynx (Waters). *Trans-4*-OH-PZQ d5 was used as an internal standard also for *cis*-OH-PZQ since their retention times were similar. Quality control samples at 9.8, 78.1, and 1250 ng/mL were injected at regular intervals during each analysis.

### *CYP3A4*,* CYP3A5*, *CYP2C19*, *CYP2C9* and *CYP2J2* genotyping

Genomic DNA was extracted from whole-blood samples using QIAamp DNA MidiKit (QIAGEN GmbH, Hilden, Germany) as per the manufacturer’s protocol. The purity and quantity of the extracted DNA were measured using NanoDrop 2000 (Thermo Scientific, Saveen Warner, Sweden). Genotyping for the common functional variant alleles in genes coding for CYP enzymes relevant for PZQ disposition was performed using allelic discrimination TaqMan genotyping assays (Applied Biosystems, CA, USA) as previously discussed^20,31^.

Briefly, genotyping was performed for *CYP3A4 * (*1B), *CYP3A5 * (*3, *6), *CYP2C19 * (*2, *3, *17), *CYP2C9 * (*2, *3), and *CYP2J2 * (*7) as described previously^20,31^. Genotyping for CYP3A5*7 was not done as previous studies reported its absence in the Ethiopian population^17,32^. The allelic discrimination reactions were performed using TaqMan® genotyping assays (Applied Biosystems®, CA, USA) with the following ID numbers for the (SNPs): C__11711730_20 for CYP3A4*1B (− 392A > G, rs2740574), C__26201809_30 for CYP3A5*3 (c.6986A4G, rs776746), C__30203950_10 for CYP3A5*6 (g.14690G4A, rs10264272), C__25986767_70 for CYP2C19*2 (rs4244285), C__2,7861809_10 for CYP2C19*3 (rs4986893), C__469857_10 for CYP2C19*17 (rs12248560), C__25625805_10 for CYP2C9*2 (rs1799853), C__27104892_10 for CYP2C9*3 (rs1057910) and C_9581699_80 for CYP2J2 *7.

The genotyping was performed on Applied Biosystems^®^ 7500 Real-Time PCR Systems (Applied Biosystems, United States). The final volume of the PCR mixture was 10 μL, consisting of 5μL TaqMan fast advanced master mix (Applied Biosystems, United States), 3.5 μL deionized water, 0.5 μL 20× drug metabolizing genotype assay mix and 1 μL genomic DNA. The thermal cycler condition involves an initial step at 60 °C for 30 s, hold stage at 95 °C for 10 min, followed by PCR stage for 40 cycles of 95 °C for 15 s, 60 °C for 1 min and after reading stage with 60 °C for 30 s.

### Study outcomes

The study outcomes were praziquantel, *trans-* and *cis-4-OH-PZQ*, metabolic ratios (*trans*-4-hydroxy-praziquantel/praziquantel and *cis*-4-hydroxy-praziquantel/praziquantel) concentrations and the effect of *CYP3A4*, *CYP3A5*, *CYP2C19*, *CYP2C9* and *CYP2J2* genotypes as well as non-genetic factors on the plasma concentrations.

### Statistical data analysis

Baseline socio-demographic, clinical, and laboratory parameters were described using means and standard deviations (SD) or medians and interquartile range (IQR) for continuous variables and as percentages for categorical variables. The Chi-square test was used to compare the genotype and allele frequencies between the observed and expected according to the Hardy–Weinberg equilibrium.

The plasma concentration data was converted to log_10_ values before statistical analysis. Independent sample t-tests or One-way ANOVA was used to compare the log-plasma PZQ, *trans*-4-OH-PZQ, and *cis*-4-OH-PZQ concentrations as well as the metabolic ratios (*trans*-4-OH-PZQ/PZQ and *cis*-4-OH-PZQ/PZQ) across the different CYP450 genotypes. Then, the arithmetic means of the log-transformed PZQ, *trans*-4-OH-PZQ, *cis*-4-OH-PZQ, *trans*-4-OH-PZQ/PZQ, and *cis*-4-OH-PZQ/PZQ concentrations were anti-logged to obtain their respective geometric means (GM) and standard deviations (SD).

A Univariate analysis followed by a multivariate linear regression analysis was performed to identify predictors of PZQ, *trans*-4-OHPZQ, and *cis*-4-OH-PZQ plasma concentrations and metabolic ratios. Variables age, sex, baseline *S. mansoni* infection status, Soil-Transmitted Helminths (STH) infection, *S. mansoni*–STH co-infection, anemia status, HAZ, BAZ, and the CYP450 genotypes were tested in the linear regression analysis. Variables with *p*-value < 0.2 from the univariate analysis were included in the multivariate analysis. In all the analyses, p-values were two-sided, and p < 0.05 was considered statistically significant. Statistical analyses were performed using Statistical Package for Social Sciences (SPSS) software for Windows version 24 (SPSS, IBM Corp, Armonk, NY, USA).

## Result

### Socio-demographic characteristics

A total of 446 school children who received the standard PZQ and ALB preventive chemotherapy through MDA to control schistosomiasis and soil-transmitted helminths participated in this study. The median age of the participants was 11 years (IQR = 8–13). Female participants were 51.6%. The proportions of participants with stunting and wasting were 21.9% and 9.3%, respectively. Only 4.3% of the participants were anemic at baseline assessment, as presented in Table 1.

**Table 1:** Baseline characteristics of the study population.

Variable	Category	N (%)
Age (years)	Median (Q1, Q3)	11 (8, 13)^a^
	≤ 12 years	331 (74.2)
	> 12 years	115 (25.8)
Sex	Female	230 (51.6)
	Male	216 (48.4)
*S. mansoni*	Infected	319 (87.4)
	No infection	127 (28.5)
STH infection	Infected	304 (65.9)
	No infection	142 (30.8)
*S. mansoni* and STH co-infection	Co-infected	197 (44.2)
	No co-infection	249 (55.8)
Weight (kg)	Median (IQR)	31.5 (26.0–39.8)^a^
Height (cm)	Median (IQR)	138.0 (128.0–150.0)^a^
BMI (kg/m2)	Median (IQR)	15.0 (17.0–18.8)^a^
Stunting status (HAZ)	Stunted	87 (21.9)
	Not stunted	310 (78.1)
Wasting status (BAZ)	Wasted	37 (9.3)
	Not wasted	360 (90.7)
Haemoglobin concentration	Median (IQR)	13.9 (13.0–15.0)^a^
Anaemia status	Anaemic	17 (4.3)
	Not anaemic	377 (95.7)

*SD* standard deviation, *IQR* interquartile range, *BAZ* body mass index (BMI) for age Z score, *HAZ* height for age Z score.

^a^N (%) not applicable.

### Genotypes and alleles frequencies

There were no significant differences between the observed and expected genotype frequencies according to the Hardy Weinberg Equilibrium. The highest allele frequency was observed for CYP3A5*3 (48.5%) followed by CYP3A4*1B (40.4%), and the lowest allele frequency was 1.6% for CYP2C9*2 (Table 2).

**Table 2:** Genotypes and allele frequencies for *CYP3A4*, *CYP3A5*, *CYP2C19*, *CYP2C9* and *CYP2J2.*

Genotype		Frequency N (%)
CYP3A4*1B (−*392A* > *G*)	*1/*1	158 (35.7)
	*1/*1B	212 (47.9)
	*1B/*1B	73 (16.5)
CYP3A5*3c.6986A > *G*	*1/*1	120 (27.1)
	*1/*3	216 (48.8)
	*3/*3	107 (24.1)
CYP3A5*6c.14690G > *A*	*1/*1	332 (74.9)
	*1/*6	94 (21.2)
	*6/*6	17 (3.8)
CYP2C19*2	*1/*1	364 (82.2)
	*1/*2	73 (16.5)
	*2/*2	6 (1.4)
CYP2C19*3	*1/*1	427 (96.4)
	*1/*3	16 (3.6)
	*3/*3	0 (0.0)
CYP2C19*17	*1/*1	298 (67.3)
	*1/*17	129 (29.1)
	*17/*17	16 (3.6)
CYP2C9*2	*1/*1	431 (97.3)
	*1/*2	10 (2.3)
	*2/*2	2 (0.5)
CYP2C9*3	*1/*1	418 (94.4)
	*1/*3	25 (5.6)
	*3/*3	0 (0.0)
CYP2J2*7	*1/*1	336 (75.8)
	*1/*7	96 (21.7)
	*7/*7	11 (2.5)

Allele	Minor allele	Percentage
CYP3A4*1B (−*392A* > *G*)	*1B	40.4
CYP3A5*3c.6986A > *G*	*3	48.5
CYP3A5*6c.14690G > *A*	*6	14.4
CYP2C19*2	*2	9.6
CYP2C19*3	*3	3.6
CYP2C19*17	*17	18.2
CYP2C9*2	*2	1.6
CYP2C9*3	*3	2.8
CYP2J2*7	*7	13.3

### Effect of CYP genotypes on PZQ concentration and metabolic ratios

For assessing the effect of CYP genotypes on PZQ plasma concentration, its major metabolites, and respective metabolic ratios, we categorized all genotypes as extensive metabolizers (*1/*1) and carriers of defective variant allele (intermediate or slow metabolizers) except for *CYP2C19* genotype. *CYP2C19* genotype was categorized as extensive and ultra-rapid metabolizers (*1/*1, *17 carriers), intermediate and slow metabolizers (*2, *3 carriers). The geometric means of PZQ, *trans-*4-OH PZQ/PZQ and *cis-*4-OH PZQ/PZQ among the different genotype groups are summarized in Table 3. The overall geometric means ± SDs of PZQ, *trans-*4-OH PZQ and *Cis-*4-OH PZQ, were 221.7 ± 5.6 ng/mL, 4406.2 ± 2.7 ng/mL and 220.0 ± 3.8 ng/mL, respectively. PZQ plasma concentrations were significantly associated with *CYP2C19* and *CYP2J2* genotypes. Likewise, *CYP2C19* and *CYP2J2* genotypes had significant association with the metabolic ratios both *trans-*4-OH PZQ/PZQ and *cis-*4-OH PZQ/PZQ.

**Table 3:** Comparison of the GMs of PZQ, *trans-*4-OH-PZQ/PZQ, and *cis*-4-OH-PZQ/PZQ between CYP450 genotypes using One-way ANOVA (GM—Geometric mean, SD- Standard deviation).

Genotype		N	Plasma concentrations					
			PZQ GM ± SD (ng/mL)	*p-*value	*trans*-4-OHPZQ/PZQ (GM ± SD)	*p-*value	1000* *cis*-4-OHPZQ/PZQ (GM ± SD)	*p-*value
*CYP3A4*	*1/*1	154	213.3 ± 5.8	0.73	19.7 ± 2.9	0.92	1022.0 ± 2.1	0.58
	*1B carriers	273	226.6 ± 5.5		20.3 ± 2.9		976.2 ± 2.4	
*CYP3A5*	*1/*1	61	225.7 ± 5.9	0.93	19.3 ± 2.9	0.80	984.6 ± 2.0	0.94
	*3 or *6 carriers	366	221.1 ± 5.6		20.0 ± 2.9		993.8 ± 2.3	
*CYP2C19*	*1/*1, *17 carriers	336	190.8 ± 5.8	< 0.001	23.0 ± 2.9	< 0.001	1100.4 ± 2.3	< 0.001
	*2 or *3 carriers	91	385.8 ± 4.2		11.6 ± 2.3		677.8 ± 2.0	
*CYP2C9*	*1/*1	391	222.3 ± 5.4	0.91	20.1 ± 2.8	0.41	997.3 ± 2.2	0.69
	*2, *3 carriers	36	214.9 ± 7.6		17.2 ± 3.5		941.3 ± 2.4	
*CYP2J2*	*1/*1	324	242.8 ± 5.0	0.052	18.5 ± 2.7	0.01	939.7 ± 2.2	0.01
	*7 carriers	103	166.5 ± 7.3		24.8 ± 3.4		1178.5 ± 2.3	

### Predictors of praziquantel plasma concentration

To identify predictors of PZQ plasma concentration, we analyzed log10 converted concentration data using univariate and multivariate linear regression. Variables with *p-*value ≤ 0.2 in the univariate analysis, such as sex, wasting, *CYP2C19* and *CYP2J2* genotypes, were included in the multivariate analysis (Table 4). Sex and *CYP2C19* remained significant predictors of PZQ plasma concentration in the multivariate analysis. Male participants had a mean increase in concentration of 0.27 ng/ml (95% CI,0.12 – 0.42). Likewise, carriers of *CYP2C19 * (*2, *3) had an increase in mean PZQ plasma concentration of 0.27 ng/ml (95% CI 0.08–0.46 and p = 0.005) compared to CYP2C19 *1/*1, or *17 carriers.

**Table 4:** Univariate and multivariate linear regression analysis for predictors of PZQ plasma concentration.

Variable		N (%)	Univariate		Multivariate	
			Crude log mean dif. (95% CI)	*p-*value	Adjusted log mean dif. (95% CI)	*p-*value
Age (years)	≤ 12 years	317 (73.7)	1	0.44		
	> 12 years	113 (26.3)	−0.06 (−0.23 to 0.10)			
Sex	Female	223 (51.9)	1	< 0.001	1	** < 0.001**
	Male	207 (48.1)	0.27 (0.13 to 0.41)		**0.27 (0.12 to 0.42)**	
*S. mansoni* infection	No	123 (28.6)	1	0.39		
	Yes	307 (71.4)	−0.07 (−0.23 to 0.09)			
STH infection	No	140 (32.6)	1	0.97		
	Yes	290 (67.4)	−0.003 (−0.16 to 0.15)			
*S. mansoni*– STH co-infection	No	226 (54.9)	1	0.69		
	Yes	186 (45.1)	−0.03 (−0.18 to 0.12)			
Anemia status	Non anemic	361 (95.0)	1	0.88		
	Anemic	19 (5.0)	−0.03 (−0.38 to 0.32)			
Stunting	Normal	298 (77.6)	1	0.51		
	Stunted	86 (22.4)	0.06 (−0.12 to 0.24)			
Wasting	Normal	347 (90.4)	1	0.07	1	0.12
	Wasted	37 (9.6)	0.24 (−0.02 to 0.49)		0.19 (−0.06 to 0.45)	
*CYP3A4*	*1/*1	154 (36.1)	1	0.73		
	*1B carriers	273 (63.9)	0.03 (−0.12 to 0.18)			
*CYP3A5*	*1/*1	61 (14.3)	1	0.93		
	*2 or *3 carriers	366 (85.7)	−0.01 (−0.21 to 0.19)			
*CYP2C19*	*1/*1, or *17 *carriers*	336 (78.7)	1	< 0.001	**1**	**0.005**
	*2 or *3 carriers	91 (21.3)	0.31 (0.13 to 0.48)		**0.27 (0.08 to 0.46)**	
*CYP2C9*	*1/*1	391 (91.6)	1	0.91		
	*2 or *3 carriers	36 (8.4)	−0.02 (−0.27 to 0.24)			
*CYP2J2*	*1/*1	324 (75.9)	1	0.05	1	0.30
	*7 carriers	103 (24.1)	−0.16 (−0.33 to 0.002)		−0.10 (−0.27 to 0.08)	

Significant values are in bold.

### Predictors of *trans*-4-OH-PZQ/PZQ metabolic ratio

We used univariate and multivariate linear regression analysis to assess predictors of trans-4-OH-PZQ/PZQ. In the univariate analysis, sex, wasting, *CYP2C19,* and *CYP2J2* genotypes had *p-values* ≤ 0.2 and were included in the multivariate model. In the multivariate analysis, sex and *CYP2C19* genotype were significant factors predicting *trans*-4-OH-PZQ/PZQ. Male participants had a mean decrease of *trans*-4-OH-PZQ/PZQ metabolic ratio by 0.15 ng/ml (95% CI −0.23 to −0.06) compared to female participants. Similarly, CYP2C19*2 or *3 carriers had significantly lower mean *trans*-4-OH-PZQ/PZQ metabolic ratios than CYP2C19 *1/*1, or *17 carriers (Table 5).

**Table 5:** Univariate and multivariate linear regression analysis for *trans*-4-OH PZQ/PZQ metabolic ratio predictors.

Variable		N (%)	Univariate		Multivariate	
			Crude log mean dif. (95% CI)	*p*-value	Adjusted log mean dif. (95% CI)	p-value
Age (years)	≤ 12 years	317 (73.7)	1	0.72		
	> 12 years	113 (26.3)	0.02 (−0.08 to 0.12)			
Sex	Female	223 (51.9)	1	< 0.001	1	0.006
	Male	207 (48.1)	−0.15 (–0.23 to −0.06)		−0.13 (−0.22 to −0.04)	
*S. mansoni* infection	No	123 (28.6)	1	0.84		
	Yes	307 (71.4)	0.01 (−0.09 to 0.11)			
STH infection	No	140 (32.6)	1	0.99		
	Yes	290 (67.4)	−0.001 (−0.09 to 0.09)			
*S. mansoni*–STH co-infection	No	226 (54.9)	1	0.96		
	Yes	186 (45.1)	−0.002 (−0.09 to 0.09)			
Anemia status	Non anemic	361 (95.0)	1	0.46		
	Anemic	19 (5.0)	−0.08 (−0.30 to 0.13)			
Stunting	Normal	298 (77.6)	1	0.52		
	Stunted	86 (22.4)	−0.04 (−0.15 to 0.08)			
Wasting	Normal	347 (90.4)	1	0.19	1	0.39
	Wasted	37 (9.6)	−0.11 (−0.27 to 0.05)		−0.07 (−0.23 to 0.09)	
*CYP3A4*	*1/*1	154 (36.1)	1	0.92		
	*1B carriers	273 (63.9)	0.005 (−0.09 to 0.10)			
*CYP3A5*	*1/*1	61 (14.3)	1	0.80		
	*3 or *6 carriers	366 (85.7)	0.02 (−0.11 to 0.14)			
*CYP2C19*	*1/*1, or *17 carriers	336 (78.7)	1	< 0.001	1	< 0.001
	*2 or *3carriers	91 (21.3)	−0.30 (−0.40 to −0.19)		−0.29 (−0.41 to −0.18)	
*CYP2C9*	*1/*1	391 (91.6))	1	0.41		
	*2 or *3carriers	36 (8.4	−0.07 (−0.22 to 0.09)			
*CYP2J2*	*1/*1	324 (75.9)	1	0.01	1	0.14
	*7 carriers	103 (24.1)	0.13 (0.03 to 0.23)		0.08 (0.03 to 0.19)	

### Predictors of *cis*-4-OH-PZQ/PZQ

In the univariate analysis, sex, *S. mansoni* infection status, *CYP2C19,* and *CYP2J2* genotypes had *p-values* ≤ 0.2 and were included in the multivariate model. In the multivariate analysis, sex, *CYP2C19,* and *CYP2J2* genotype remained significant predictors of *cis*-4-OH-PZQ/PZQ metabolic ratio. In the multivariate model, sex, *CYP2J2* and *CYP2C19* genotypes were significant predictors of *cis*-4-OH-PZQ/PZQ metabolic ratio*. CYP2C19 * (*1, *17) and CYP2J2*7 carriers had higher *cis*-4-OH-PZQ/PZQ metabolic ratio compared to *CYP2C19 * (*2, *3) carriers and CYP2J2*1/*1 genotype (p   < 0.001 and 0.038) respectively (Table 6).

**Table 6:** Univariate and multivariate linear regression analysis for predictors of *cis*-4-OH-PZQ/PZQ.

Variables		Crude, N (%)	Univariate		Multivariate	
			Crude log mean dif. (95% CI)	*p*-value	Adjusted log mean dif. (95% CI)	p-value
Age (years)	≤ 12 years	317 (73.7)	1	0.31		
	> 12 years	113 (26.3)	−0.04 (−0.12 to 0.04)			
Sex	Female	223 (51.9)	1	< 0.001	1	0.001
	Male	207 (48.1)	−0.12 (−0.18 to −0.05)		−0.11 (−0.18 to −0.04)	
*S. mansoni* infection	No	123 (28.6)	1	0.09		0.12
	Yes	307 (71.4)	0.06 (−0.01 to 0.14)		0.06 (−0.02 to 0.13)	
STH infection	No	140 (32.6)	1	0.27		
	Yes	290 (67.4)	−0.04 (−0.11 to 0.03)			
*S. mansoni and* STH co-infection	No	226 (54.9)	1	0.85		
	Yes	186 (45.1)	0.01 (−0.06 to 0.08)			
Anemia status	Non anemic	361 (95.0)	1	0.56		
	Anemic	19 (5.0)	−0.05 (−0.21 to 0.12)			
Stunting	Normal	298 (77.6)	1	0.40		
	Stunted	86 (22.4)	−0.04 (−0.13 to 0.05)			
Wasting	Normal	347 (90.4)	1	0.56		
	Wasted	37 (9.6)	−0.04 (−0.16 to 0.09)			
*CYP3A4*	*1/*1	154 (36.1)	1	0.58		
	*1B carriers	273 (63.9)	−0.02 (−0.09 to 0.05)			
*CYP3A5*	*1/*1	61 (14.3)	1	0.94		
	*3 or *6 carriers	366 (85.7)	0.004 (−0.09 to 0.10)			
*CYP2C19*	*1/*1 or *17 *carriers*	336 (78.7)	1	< 0.001	1	< 0.001
	*2 or *3 carriers	91 (21.3)	−0.21 (−0.29 to −0.13)		−0.19 (−0.27 to −0.11)	
*CYP2C9*	*1/*1	391 (91.6)	1	0.68		
	*2 or *3 carriers	36 (8.4)	−0.03 (−0.0.15 to 0.10)			
*CYP2J2*	*1/*1	324 (75.9)	1	0.01	1	0.038
	*7 carriers	103 (24.1)	0.10 (0.02 to 0.18)		0.08 (0.01 to 0.16)	

## Discussion

Schistosomiasis remains a major problem in Sub-Saharan Africa, and millions of children living in endemic countries receive periodic school-based mass praziquantel administration to control and halt transmission. Genetically polymorphic CYP enzymes metabolize PZQ, but the impact of genetic variations on drug exposure, particularly in black Africans, the most genetically diverse population, is not well explored. We investigated the effect of genetic variations on plasma concentrations of PZQ, its major metabolites (*trans-*4-OH PZQ, *Cis-*4-OH PZQ), and metabolic ratios (*trans-*4-OH PZQ/PZQ and *Cis-*4-OH PZQ/PZQ) among school children who received mass PZQ and ALB administration.

There are several notable findings from this study: First, there was a significant association of *CYP2C19* and *CYP2J2* genotypes with PZQ plasma concentration and *trans-*4-OH PZQ/PZQ and *Cis-*4-OH PZQ/PZQ metabolic ratios. Carriers of defective *CYP2C19* variant alleles (*2 or *3) had significantly higher PZQ plasma concentration and lower metabolic ratio than *CYP2C19* extensive metabolizers. On the other hand, CYP2J2*7 carriers had a borderline lower PZQ plasma concentration and significantly higher cis*-*4-OH PZQ/PZQ metabolic ratio. Second, sex significantly predicted PZQ plasma concentration and *trans-*4-OH PZQ/PZQ and *Cis-*4-OH PZQ/PZQ metabolic ratios. Male participants had higher PZQ plasma concentration and lower *trans-*4-OH PZQ/PZQ and *Cis-*4-OH PZQ/PZQ metabolic ratios compared to females. No significant effect of having infection by *S. mansoni* or soil-transmitted helminths, or nutritional status (stunting, wasting) or *CYP3A4, CYP3A5*, or *CYP2C9* genotype on plasma praziquantel concentration or its metabolic ratio was observed. This is the first study to explore the impact of genetic and non-genetic factors, including *S. mansoni* and soil-transmitted helminth infection status in Ethiopian children.

In humans, praziquantel is metabolized primarily by *CYP2C19* to 4-OH PZQ, the major metabolite, and to some extent by *CYP1A2, CYP3A4, CYP3A5*, and *CYP2C9*^19,20,25^. Genes coding for these metabolizing enzymes are genetically polymorphic. Thus, investigating the effect of genetics on PZQ exposure is important, especially in the genetically diverse SSA population^20,21^, and variations in treatment outcomes have been reported^13^. Moreover, the African continent contributes more than 90% of the global disease burden^4^, and Preventive chemotherapy through MDA campaigns periodically without prior diagnosis to all at-risk children living in endemic areas. The genotype and allele frequencies of CYP3A4*1B, *CYP3A5 * (*3,*6), *CYP2C9 * (*2,*3), *CYP2C19 * (*2,*3,*17)*,* and CYP2J2*7 observed in our study were similar with those of previous studies conducted in Ethiopian population^32–34^.

In addition to exploring the impact of genotype in relevant enzymes involved in the intricate metabolic pathway of praziquantel, we investigated the role of *CYP2J2* genotype, which has not been previously examined in this context. It is noteworthy that albendazole, co-administered with praziquantel in school-based MDA programs as per the WHO recommendations in endemic areas, is primarily metabolized by CYP2J2^35,36^. With our study participants receiving both praziquantel and albendazole as part of preventive chemotherapy, we aimed to evaluate any potential impact of *CYP2J2* genotype. Drug interactions could potentially affect the relationship between genotype and plasma concentrations of co-administered drugs, necessitating further investigation. Furthermore, the relevance of the CYP2J2 enzyme for praziquantel metabolism has not been previously explored. Our finding of a significant impact of *CYP2J2* genotype on praziquantel plasma concentration could be attributed to its influence on albendazole metabolism, potentially altering the absorption, metabolism, and transport of praziquantel. Alternatively, CYP2J2 may directly affect praziquantel metabolism, although this aspect requires further investigation.

Our study found a significant association between *CYP2C19* genotype and PZQ plasma concentration; a higher PZQ concentration was observed among children carrying defective alleles *CYP2C19 * (*2 or *3) compared to those with wild type (CYP2C19 *1/*1)) or CYP2C19 *17 carriers. Likewise, the *CYP2C19* genotype was also significantly associated with *trans* 4-OH-PZQ/PZQ and *cis* 4-OH-PZQ/PZQ) metabolic ratios. *CYP2C19* extensive metabolizers had lower metabolic ratios compared to those carrying defective *CYP2C19* alleles variant allele (*2 or *3)*.* Similar findings were reported among *S. mansoni*-infected Tanzanian children who received praziquantel therapy, where the *CYP2C19* genotype was significantly associated with PZQ plasma concentration and the *trans* 4-OH-PZQ/PZQ metabolic ratio^20^. However, the study in Tanzania did not assess *cis*-OH-PZQ/PZQ as we did in this study. This finding strengthens the previous evidence that *CYP2C19* is a major metabolic pathway for the formation of major PZQ metabolites such as *trans*-and *cis* 4-OH-PZQ^20,25,37^. Furthermore, these findings indicate the relevance of the *CYP2C19* genotype in determining praziquantel plasma exposure among children receiving PZQ MDA. However, the relevance of *CYP2C19* genotype for the safety and efficacy of praziquantel needs to be investigated.

Our study also found a borderline association between *CYP2J2* genotype (p = 0.05) and PZQ plasma concentration, where higher PZQ plasma concentrations were observed among CYP2J2 *1/*1 genotypes compared to CYP2J2 *7 carriers. *CYP2J2* genotype was significantly associated with both *trans*-4-OH-PZQ/PZQ and *cis* 4-OH-PZQ/PZQ) the metabolic ratios, being higher among CYP2J2 *7 carriers than the wild type (CYP2J2 *1/*1). The CYP2J2 *7 variant allele is reported to be associated with increased enzyme activity^34^, which is in line with our findings. A recent study reported the contribution of *CYP2J2* genotype for PZQ metabolism though not statistically significant^31^. The significant association of *CYP2J2* genotype with variability in PZQ plasma concentration in our study may indicate the contribution of *CYP2J2* enzyme for PZQ metabolism. CYP2J2 metabolizes structurally diverse compounds that are also metabolized by *CYP3A4*, but with differences in regioselectivity^35,38^. In addition to its role in endogenous metabolism, recent studies highlighted the importance of *CYP2J2* for metabolizing various drugs, including anti-malarial and anti-tuberculosis that are widely used in Africa^39,40^. For the first time, our result indicates *CYP2J2* genotype plays an important role in inter-individual variability in PZQ and its metabolites exposure.

Our study also found significant sex differences in PZQ and its metabolite exposure. Male participants had significantly higher PZQ plasma concentration and lower *trans-*4-OH PZQ/PZQ and *cis-*4-OH PZQ/PZQ metabolic ratios than females. This observation aligns with previous research indicating higher CYP enzyme activity among females compared to males^41,42^. Consistent with these findings, our study suggests that females displayed lower PZQ plasma concentrations, implying enhanced enzyme activity. Sex differences in drug metabolism stem from a range of biological factors, including variations in hormone levels, enzyme expression, body weight, body mass index (BMI), fat distribution, and other physiological disparities between males and females^43,44^. These differences can lead to variations in drug absorption, distribution, metabolism, and elimination within the body, thereby resulting in distinct pharmacokinetic profiles between males and females.

Although *CYP3A4, CYP3A5,* and *CYP2C9* enzymes were identified as relevant for the metabolism of PZQ in previous studies^18,19,25,26^, our study found no significant association between *CYP3A4*, *CYP3A5,* and *CYP2C9* genotypes and PZQ plasma concentration, and its metabolic ratios. This finding further suggests that *CYP2C19* is a major metabolic pathway for the formation of major PZQ metabolites in humans. In addition, our study identified the *CYP2J2* genotype as another possible pathway for PZQ metabolism.

## Conclusion

We conclude that plasma concentrations of PZQ and its metabolic ratio display wide inter-individual variability, partly due to pharmacogenetic variations and sex differences. *CYP2C19* and *CYP2J2* genotypes significantly predict PZQ plasma concentration and its metabolic ratios -*trans* 4-OH-PZQ/PZQ and *Cis* 4-OH-PZQ/PZQ. *CYP2J2* genotype is significantly associated with PZQ and its major metabolites exposure; therefore, *CYP2J2* could be another important pathway for PZQ metabolism in humans. This study highlights the importance of pharmacogenetic variation for PZQ pharmacokinetics. The impact of genetic variations on PZQ treatment outcomes requires further investigations.

## Data Availability

All relevant data are included in the manuscript; further enquiries can be directed to the corresponding author.
